# Control angiography for perioperative myocardial Ischemia after coronary surgery: meta-analysis

**DOI:** 10.1186/s13019-018-0710-0

**Published:** 2018-02-27

**Authors:** Fausto Biancari, Vesa Anttila, Angelo M. Dell’Aquila, Juhani K. E. Airaksinen, Debora Brascia

**Affiliations:** 1 Department of Surgery, University of Turku, Turku, Finland; 2 Department of Surgery, University of Oulu, Oulu, Finland; 3 Heart Center, Turku University Hospital and University of Turku, Hämeentie 11, 20521 Turku, PL 52 Finland; 4 Department of Cardiac Surgery, University Hospital, Münster, Germany

**Keywords:** Coronary artery bypass, Perioperative myocardial infarction, Angiography, Percutaneous coronary intervention

## Abstract

**Background:**

Perioperative myocardial ischemia (PMI) in patients undergoing coronary artery bypass grafting (CABG) is associated with poor outcome. The aim of this study was to pool the available data on the outcome after control angiography and repeat revascularization in patients with perioperative myocardial ischemia (PMI) after coronary artery bypass grafting (CABG).

**Methods:**

A literature review was performed through PubMed, Scopus, ScienceDirect and Google Scholar to identify studies published since 1990 evaluating the outcome of PMI after CABG.

**Results:**

Nine studies included 1104 patients with PMI after CABG and 1056 of them underwent control angiography early after CABG. Pooled early mortality after reoperation for PMI without control angiography was 43.6% (95%CI 29.7-57.6%) and 79.8% of them (95%CI 64.4-95.2%) had an acute graft failure detected at reoperation. Among patients who underwent control angiography for PMI, 31.7% had a negative finding at angiography (95%CI 25.6-37.8%) and 62.1% had an acute graft failure (95%CI 56.6-67.6%). Repeat revascularization was performed after early control angiography in 46.3% of patients (95%CI 39.9-52.6%; 54.2% underwent repeat surgical revascularization; 45.8% underwent percutaneous coronary intervention). Pooled early mortality after control angiography with or without repeat revascularization was 8.9% (95%CI 6.7-11.1%). Three studies reported on early mortality rates which did not differ between repeat surgical revascularization and PCI (11.7% vs. 9.2%, respectively; risk ratio 1.45, 95%CI 0.67-3.11). In these three series, early mortality after conservative treatment was 5.9% (95%CI 3.6-8.2%).

**Conclusions:**

Control angiography seems to be a valid life-saving strategy to guide repeat revascularization in hemodynamically stable patients suffering PMI after CABG.

## Background

Perioperative myocardial ischemia (PMI) in patients undergoing coronary artery bypass grafting (CABG) is associated with poor outcome [1–3]. Still, a reliable and clinically useful definition of this condition remains elusive and with it also the optimal treatment of patients suffering PMI [1]. Recently, the European Society of Cardiology endorsed a document suggesting new cutoff values for cardiac troponin levels for the diagnosis of PMI after CABG and proposed a treatment strategy for its treatment [1]. This document recognized the clinical importance of PMI in these patients, however it unclear whether postoperative control angiography and, when needed, prompt repeat revascularization is beneficial in these patients. We sought to investigate this issues by pooling the available data from the literature on the outcome of patients with PMI after CABG.

## Methods

The present systematic review and meta-analysis is registered in the International prospective register of systematic reviews PROSPERO with the reference code CRD (ID=CRD42017076614).

### Search Strategy

The guidelines for Preferred Reporting Items for Systematic reviews and Meta-Analyses (PRISMA) were applied [4]. A literature review was performed through PubMed, Scopus, ScienceDirect and Google Scholar on September 2017, to identify any study being published since 1990 evaluating the outcome of patients who underwent angiography and any treatment for PMI immediately after CABG. The retrieval terms were “perioperative myocardial ischemia”, “perioperative myocardial infarction”, “angiography” combined with “cardiac surgery” or “coronary artery bypass”. The abstracts of retrieved studies were scrutinized and each study was independently evaluated by two investigators (D.B, F.B.) for inclusion or exclusion from this analysis. Reference lists of retrieved articles were examined as well to identify any article of fulfilling the pre-specified inclusion criteria.

### Treatment Definition and Inclusion/Exclusion Criteria

Studies eligible for the present studies were those reporting on perioperative myocardial ischemia after CABG. Studies that met the Population, Interventions, Comparison and Outcomes (PICO) criteria (Tab. 1) were included in the present meta-analysis.

**Table 1:** Participants, intervention, comparison and outcomes (PICO) of the present meta-analysis

PICO	Description
Population	Patients who developed perioperative myocardial ischemia immediately after coronary artery bypass grafting
Intervention	Coronary angiography and repeat myocardial revascularization
Comparison	None
Outcomes	In-hospital/30-day mortality

To enter this analysis, studies had to fulfil all these inclusion criteria: (1) provide data on patients who suffered PMI immediately after CABG according to the investigators’ definition criteria; (2) include patients undergoing CABG as isolated procedure or associated with any other major cardiac procedure; (3) include patients aged 18 years or older; (4) be a prospective or retrospective observational study; (5) be published in English language as a full article; (6) include at least 10 patients with postoperative PMI; and (7) be published since 1990.

Articles were not eligible for study inclusion in case of (1) angiography and/or repeat revascularization performed after discharge from the hospital associated with the index procedure; (2) ambiguous or inaccurate data; (3) lack on information on in-hospital/30-day mortality; (4) data reported only in abstracts; (5) article published in non-English language.

### Data Extraction

Data was independently collected by two investigators (D.B, F.B.) and any disagreement on retrieved data was settled by consensus between these investigators. Specific or missing data were not asked to the authors of the original studies. The following data was collected in to a dedicated datasheet: first author, year of publication, study period, overall number of CABG procedures performed during the study period, type of primary procedure, number of patients with PMI, number of patients who underwent immediate control angiography, findings at angiography, type of repeat revascularization and in-hospital/30-day mortality. The quality of the included studies was assessed by two investigators (F.B., D.B.) using the National Heart, Lung, and Blood Institute (NHLBI) criteria for study quality assessment of case series (https://www.nhlbi.nih.gov/health-pro/guidelines/in-develop/cardiovascular-risk-reduction/tools/case_series; accessed on September 17, 2017).

### Outcomes

The primary outcome of this study was in-hospital/30-day postoperative mortality. Acute graft failure (defined as stenotic or occluded grafts, or faulty site of anastomosis), incomplete revascularization and new lesions of the native coronary arteries were the secondary end-points of this analysis.

### Statistical Analysis

Statistical analysis was performed using the Open Meta-Analyst software (Brown University, Providence, RI, USA; *http://www.cebm.brown.edu/openmeta/*). Means and absolute values were pooled using random effects models because of anticipated heterogeneity of the retrieved studies. The results are expressed as untransformed proportions and means with their 95% confidence intervals (CI). Heterogeneity across studies was evaluated using the I^2^ test. I^2^ <40% was considered as acceptable heterogeneity. Leave-one-out sensitivity analysis was performed to confirm consistency of the overall analysis. The impact of risk factors on in-hospital/30-day mortality was evaluated by meta-regression analysis. A *p* < 0.05 was considered statistically significant.

## Results

### Overall data

**Fig. 1:**
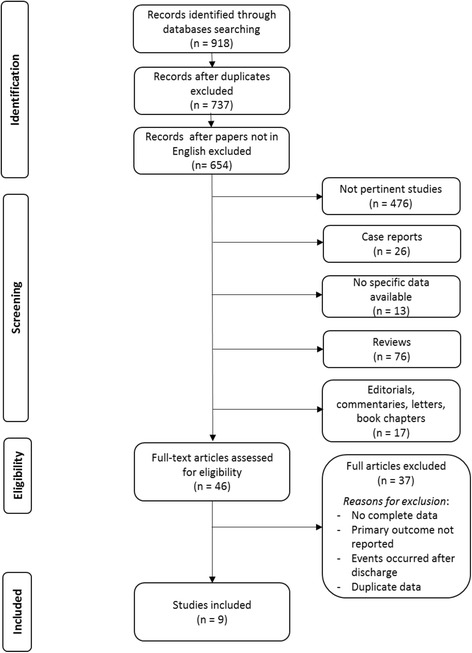
Literature search flowchart

Nine studies including 39 266 patients who underwent CABG (98.2% underwent isolated procedure) fulfilled the pre-specified selection criteria and were included in this analysis (Fig. 1). [2, 3, 5–11] Among these patients, a diagnosis of PMI was made in 1104 patients and 1056 underwent control angiography early after CABG. Angiography was performed within the same hospital stay in seven studies, within 48 hours from surgery in one study and within 24 hours from surgery in another study (Tab. 2). Definition criteria for PMI used in these studies are summarized in Table 3. All these studies reported on in-hospital or 30-day mortality after angiography followed or not by repeat revascularization. Three studies [7, 9, 11] reported on 48 patients who underwent repeat CABG without early angiography because of unstable hemodynamic conditions. Characteristics and main data of these studies are summarized in Table 1. All studies were considered of fair quality according to the NHBLI criteria.

**Table 2:** Characteristics and data of the included studies

Author	Year	Country	Type of study	Study period	NHLBI quality rating	No. of control angiography	Negative findings at angiography (%)	Graft failure at angiography (%)	Repeat revascularization (%)	Repeat CABG (%)	PCI (%)	Early mortality (%)
Rasmussen	1997	Denmark	P	1990-1995	Fair	59	27.1	72.9	45.8	45.8	0.0	5.1
Fabricius	2001	Germany	R	1999	Fair	108	41.7	58.3	39.8	31.5	8.3	9.3
Thielmann	2006	Germany	P	1999-2006	Fair	118	43.2	56.8	33.9	12.7	21.2	8.5
Karhunen	2010	Finland	R	2000-2007	Fair	23	47.8	52.2	43.5	21.7	21.7	8.7
Laflamme	2012	Canada	R	2003-2009	Fair	39	17.9	82.1	48.7	10.3	38.5	7.7
Szavits-Nossan	2012	Croatia	R	1999-2009	Fair	55	21.8	65.5	69.1	14.5	54.5	23.6
Davierwala	2013	Germany	P	2004-2010	Fair	399	36.1	61.2	41.1	32.6	8.5	7.3
Hultgren	2016	Sweden	R	2007-2012	Fair	87	31.0	59.8	44.8	28.7	16.1	11.5
Preußer	2017	Germany	R	2006-2013	Fair	168	23.2	52.4	53.6	17.9	35.7	10.7

*R* retrospective study, *P* prospective study, *NHLBI* National Heart, Lung, and Blood Institute, *CABG* coronary artery bypass grafting, *PCI* percutaneous coronary intervention

**Table 3:** Definition criteria for perioperative myocardial ischemia after coronary artery bypass grafting

Authors	Year of publication	Definition criteria
Rasmussen	1997	One or more of the following criteria:
		- new changes in the ST-segment in the ECG;
		- new Q-waves in the ECG;
		- CK-MB >80 U/L;
		- recurrent episodes of, or sustained ventricular tachyarrhythmia;
		- ventricular fibrillation;
		- hemodynamic deterioration and left ventricular failure.
Fabricius	2001	One or more of the following criteria:
		- increase in CK/CK-MB above 10%;
		- ischemic electrocardiographic episodes (new changes in ST-segment lasting at least 1 min and involving a shift from baseline of greater than or equal to 0.1 mV of ST-depression and a new postoperative Q;
		- recurrent episodes of, or sustained ventricular tachyarrhythmia or ventricular fibrillation;
		- hemodynamic deterioration despite adequate inotropic support.
Thielmann	2006	One or more of the following criteria:
		- cTnI serum level >20 ng/ml within 24 h after surgery;
		- ST-segment deviations at the J point in two or more contiguous leads with cut-off points ≥0.2 mV in leads V1, V2, or V3 and 0.1 mV in other leads or T-wave abnormalities in two or more contiguous leads as previously described;
		- hemodynamic instability despite intravenous inotropic support (>0.3 mg/kg/min).
Karhunen	2010	One or more of the following criteria:
		- ST-level changes in ECG and increased levels of cardiac biomarkers, creatine phosphokinase isoenzyme CK-MB or Tn;
		- ECG changes or findings of poor myocardial function or a new wall motion abnormality in echocardiography.
Laflamme	2012	One or more of the following criteria:
		- ECG modifications (new ST segment alterations or new Q wave);
		- refractory malignant arrhythmias;
		- elevation of cardiac biomarkers;
		- persistent low cardiac output syndrome;
		- new echocardiography wall motion abnormalities.
Szavits-Nossan	2012	One or more of the following criteria:
		- hemodynamical deterioration;
		- new ST segment depression or elevation greater than 1 mm;
		- isoenzyme ratio of creatinine phosphokinase > 0.1;
		- cardiac troponin >0.1 mmol/L;
		- sustained ventricular tachycardia;
		- repeated nonsustained ventricular tachycardia and ventricular fibrillation.
Davierwala	2013	One or more of the following criteria:
		- electrocardiographic alterations;
		- CK-MB >2x normal;
		- new regional wall motion abnormalities on echocardiography;
		- repetitive ventricular arrhythmias;
		- hemodynamic instability.
Hultgren	2016	One or more of the following criteria:
		- ECG changes;
		- chest pain;
		- Aspartate aminotransferase (ASAT) on postoperative day 1 >2.5 μkat/l;
		- Troponin T measured 3-4 days after surgery > 2000 ng/L.
Preußer	2017	One or more of the following criteria:
		- progressive postoperative elevation of cardiac enzymes;
		- ECG changes suggestive of myocardial ischemia (such as ST segment alteration);
		- major ventricular arrhythmia of unclear cause.

*ECG* electrocardiogram, *CK-MB* creatine kinase-MB, *Tn* troponin

The pooled incidence of PMI requiring angiography or immediate reintervention without control angiography was 3.5% (95%CI 0.2-6.8%, I^2^ 91%, 3 studies, 9306 patients) and the related pooled in-hospital/30-day mortality of these patients was 12.6% (95%CI 8.8-16.4%, 3 studies, 286 patients).

### Outcome of reoperation for PMI without control angiography

Pooled in-hospital/30-day mortality in patients who suffered perioperative myocardial ischemia and underwent reoperation without control angiography was 43.6% (95%CI 29.7-57.6%, I^2^ 81%, three studies, 48 patients). Among patients who underwent reoperation without control angiography, acute graft failure was detected in 79.8% of cases (95%CI 64.4-95.2%, I^2^ 49%, three studies, 48 patients).

### Outcome of PMI with control angiography

Among patients who underwent control angiography for PMI after CABG, 31.7% of them had negative finding at angiography (95%CI 25.6-37.8%, I^2^ 75%, 9 studies, 1056 patients), 62.1% of patients had an acute graft failure (95%CI 56.6-67.6%, I^2^ 66%, 9 studies, 1056 patients), 6.1% had incomplete revascularization (95%CI 0.4-12.5%, I^2^ 91%, 5 studies, 624 patients) and 3.5% has a new lesion of the native coronary arteries (95%CI 1.4-5.7%, I^2^ 91%, 9 studies, 1056 patients).

Repeat revascularization was performed after control angiography in 46.3% of patients (95%CI 39.9-52.6%, I^2^ 74%, 9 studies, 1056 patients). Among these patients, 54.2% underwent repeat surgical revascularization (95%CI 33.7-74.8%, I^2^ 97%, 9 studies, 470 patients) and 45.8% underwent any percutaneous coronary intervention (PCI) (95%CI 25.2-66.3%, I^2^ 97%, 9 studies, 470 patients). Three studies (2,3,5) reported on the graft/native artery of repeat revacularization in 294 patients, which was on the left anterior descending artery territory in 56.0% of cases (95%CI 43.2-68.8%, I^2^ 78%), on the circumflex artery territory in 21.3% of cases (95%CI 9.3-33.3%, I^2^ 84), on the right coronary artery territory in 23.3% of cases (95%CI 16.6-30.0%, I^2^ 40%) and on the diagonal arteries territory in 4.8% of cases (95%CI 0.9-8.8%, I^2^ 58%).

**Fig. 2:**
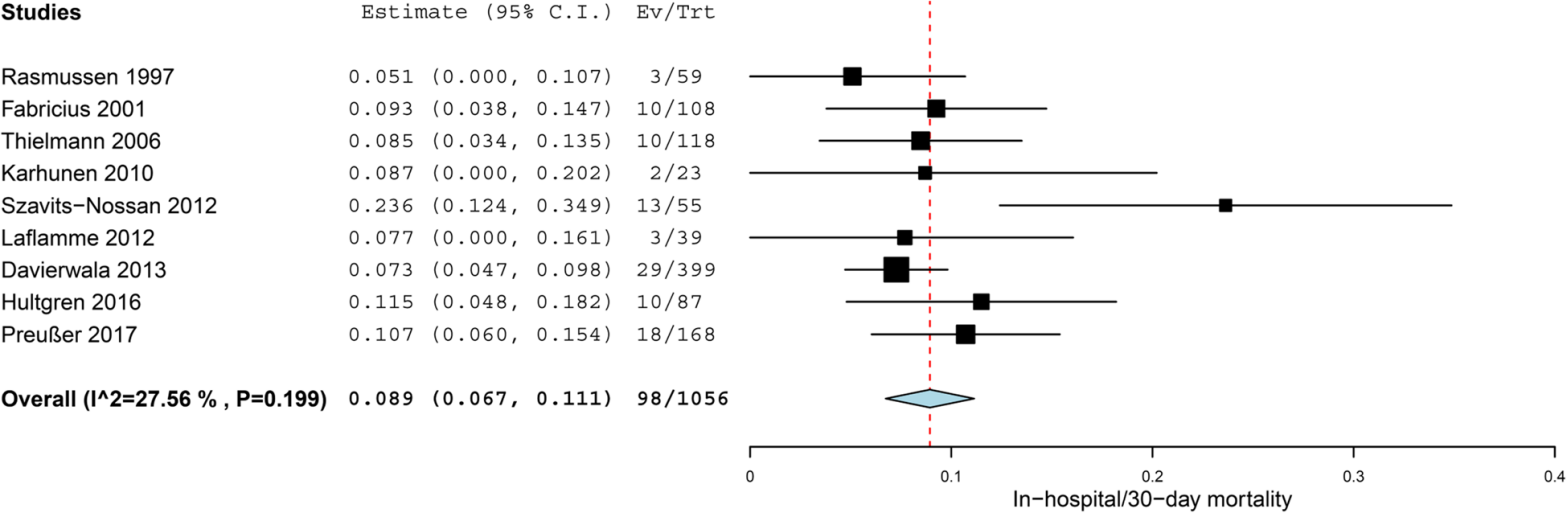
Forest plot of pooled in-hospital/30-day mortality in patients with or without perioperative myocardial ischemia after coronary artery bypass grafting

Pooled in-hospital/30-day mortality in patients who underwent control angiography was 8.9% (95%CI 6.7-11.1%, I^2^ 28%, 9 studies, 1056 patients) (Fig. 2). Three studies reported on specific early mortality after repeat surgical revascularization and PCI after control angiography without any difference between the treatment methods (CABG 11.7% vs. PCI 9.2%; risk ratio 1.45, 95%CI 0.67-3.11, I^2^ 0%, 3 studies, 175 CABG patients vs. 119 PCI patients, respectively). These three series reported on early mortality after conservative treatment as well which was 5.9% (95%CI 3.6-8.2%, I^2^ 0%, 3 studies, 391 patients), without reporting separate rates of early mortality in patients with graft failure and in those with negative angiographic findings.

## Discussion

The present pooled results suggest that severe PMI is rather uncommon, but its incidence may significantly vary between series (3.5%, 95%CI 0.2-6.8%). This estimate is also biased by the fact that most of series did not report on emergency reoperation performed without control angiography. Furthermore, the definition criteria of PMI as well as the policy of performing control angiography might vary between these series. Control angiography revealed problems of the graft or native vessels in two third of patients and it was valuable to guide a repeat revascularization in half of patients with PMI. This strategy of control angiography and, when indicated, repeat revascularization contributed to an early mortality rate of 8.9%. The lack of a control cohort with similar graft/native vessels problems treated conservatively prevents any estimation of the real benefits and possible harms of an active revascularization policy in patients with on-going ischemia after CABG. The paucity of data on angiographic findings and on the outcome of patients treated conservatively because of negative angiographic findings or lack of suitable target vessels/grafts for repeat revascularization render the interpretation of these pooled results even more difficult. However, Karhunen et al. [9] showed that a policy of control angiography in PMI patients was associated with a dramatic decrease of postoperative mortality when compared with an historical control group who did not undergo any control angiography for PMI (22.2% vs. 46.1%, p=0.015). Therefore, we may assume that in most of cases on-going severe myocardial ischemia secondary to any graft or new native vessels problem cannot be relieved by a conservative approach [6] and is associated with poor early and mid-term outcome.

The present findings indicate that control angiography may enable optimization of a reintervention strategy by identification of acute graft failure and/or new native vessels stenosis/occlusion amenable to surgical or catheter-based revascularization. Control angiography may identify also non-structural defects such as graft spam which are relievable by systemic or local administration of nitrates (9). Furthermore, angiographic findings may indicate PCI in conditions not amenable with repeat surgical revascularization. Finally, control angiography may guide toward prompt optimization of inotropic and mechanical circulatory support in patients with normal angiographic findings or poor run-off contraindicating any repeat revascularization.

This study showed that a valid and easy-to-use definition of type V myocardial infarction is needed for a prompt identification and treatment of patients with severe PMI. The present results showed that one third of patients do not have structural defects of the grafts or new stenosis/occlusions of native vessels at angiography. These patients most likely suffered of PMI because of suboptimal myocardial protection, distal coronary embolization, or subclinical myocardial injury secondary to intraoperative manipulation of the heart. The angiographic evaluation of the patients represents a unique opportunity for a practical definition of type V myocardial infarction because it may identify clinical, biochemical, electrocardiographic and echocardiographic parameters underlying PMI secondary to structural defects of the grafts and native vessels.

Control angiography for PMI has some drawbacks, which may induce clinicians to avoid prompt invasive imaging of the coronary arteries and grafts. Although coronary angiography in a patient with recent CABG may not be technically difficult, moving a patients in hemodynamically unstable conditions from the intensive care unit to the catheterization angiography laboratory can be difficult and requires major efforts from personnel. Furthermore, the use of contrast agent during control angiography may expose the patents to a significant risk of severe acute kidney injury. These important drawbacks should be weighed against the possible benefits of prompt treatment of ongoing PMI.

This meta-analysis has a number of limitations. First, the included studies are of fair quality, but do not provide all details on the characteristics and outcome of patients with PMI with negative angiographic findings and of those with graft or native vessels problems who did not undergo any repeat revascularization. Second, only three studies reported on the outcome of patients who underwent straightly a reoperation, whilst this information was not provide in the other studies. Third, we may expect that prompt recognition and treatment of PMI has a prognostic impact on the outcome of these patients, but only two study assessed the timing of angiography in this setting [2, 3]. Fourth, the included studies failed to report data on any method employed to verify intraoperatively the patency of vascular grafts [12] and whether these findings might correlate with the development of PMI. Finally, there is paucity of data comparing the impact of PCI and surgical revascularization in patients with PMI after CABG.

## Conclusions

In conclusion, control angiography seems to be a valid life-saving strategy to guide repeat revascularization in hemodynamically stable patients suffering PMI after CABG. However, a throughout assessment of the harms and benefits associated with this strategy is prevented by the lack of any possible control cohort. Further studies are needed to assess the prognostic impact of the timing of control angiography and repeat revascularization as well as to identify clinical, biochemical, electrocardiographic and echocardiographic parameters associated with PMI secondary to acute graft failure or new native vessels lesions after CABG.

## Abbreviations

PMI: perioperative myocardial infarction
CABG: coronary artery bypass grafting
PRISMA: Preferred Reporting Items for Systematic reviews and Meta-Analyses
PICO: Population, Interventions, Comparison and Outcomes
NHLBI: National Heart, Lung, and Blood Institute
CI: confidence interval
PCI: percutaneous coronary intervention
